# Sterol Regulatory Element Binding Protein (Srb1) Is Required for Hypoxic Adaptation and Virulence in the Dimorphic Fungus *Histoplasma capsulatum*

**DOI:** 10.1371/journal.pone.0163849

**Published:** 2016-10-06

**Authors:** Juwen C. DuBois, A. George Smulian

**Affiliations:** 1 Department of Pathology and Laboratory Medicine, University of Cincinnati, Cincinnati, Ohio, United States of America; 2 Cincinnati VA Medical Center, Cincinnati, Ohio, United States of America; 3 Department of Internal Medicine, University of Cincinnati, Cincinnati, Ohio, United States of America; Wadsworth Center, UNITED STATES

## Abstract

The *Histoplasma capsulatum* sterol regulatory element binding protein (SREBP), Srb1 is a member of the basic helix-loop-helix (bHLH), leucine zipper DNA binding protein family of transcription factors that possess a unique tyrosine (Y) residue instead of an arginine (R) residue in the bHLH region. We have determined that *Srb1* message levels increase in a time dependent manner during growth under oxygen deprivation (hypoxia). To further understand the role of Srb1 during infection and hypoxia, we silenced the gene encoding Srb1 using RNA interference (RNAi); characterized the resulting phenotype, determined its response to hypoxia, and its ability to cause disease within an infected host. Silencing of *Srb1* resulted in a strain of *H*. *capsulatum* that is incapable of surviving *in vitro* hypoxia. We found that without complete *Srb1* expression, *H*. *capsulatum* is killed by murine macrophages and avirulent in mice given a lethal dose of yeasts. Additionally, silencing *Srb1* inhibited the hypoxic upregulation of other known *H*. *capsulatum* hypoxia-responsive genes (HRG), and genes that encode ergosterol biosynthetic enzymes. Consistent with these regulatory functions, *Srb1* silenced *H*. *capsulatum* cells were hypersensitive to the antifungal azole drug itraconazole. These data support the theory that the *H*. *capsulatum* SREBP is critical for hypoxic adaptation and is required for *H*. *capsulatum* virulence.

## Introduction

Histoplasmosis is a disease that occurs following inhalation of air-borne *H*. *capsulatum* infectious spores or conidia[1]. As the fungus infects the host, it frequently encounters a diverse range microenvironmental conditions that can influence fungal morphology and genetic profile. The internal temperature change within the host from 25°C to 37°C induces a physical change from the mycelial form to the pathogenic budding yeast form [2]. Macrophages and dendritic cells are among the first set of cells to defend the host from attacks by the fungus. Within macrophages, yeasts cells are nutrient starved and are exposed to an unfavorable acidic environment. However, they are able to replicate and survive[3]. Therefore, because surviving varying microenvironmental stress conditions is critical for *H*. *capsulatum* pathogenesis, it must be able to adapt quickly. The *H*. *capsulatum* expression profile in response to temperature [4] nitrosative stress [5] and iron deficiency [6] has confirmed that the ability to survive environmental stressors is tightly regulated at the level of transcription. Thus, this manuscript is focused on understanding the mechanism that *H*. *capsulatum* utilizes to survive relativeoxygen deprivation (hypoxia).

In the human pathogenic fungi *Aspergillus fumigatus* [7] and *Cryptococcus neoforman*s [8,9], and in the fission yeast *Schizosaccharomyces pombe*[10,11], a sterol regulatory element binding protein (SREBP) has been identified as a key regulator of hypoxia. Fungal SREBPs control the expression of genes involved in the biosynthesis of ergosterol, lipids and heme (reviewed in [12–15]). Although the manner in which SREBP functions is similar in these fungal species, they are not identical, and much remains to be studied. Under normal oxygen and sterol conditions, the *C*. *neoformans* and *S*. *pombe* SREBP, Sre1 are membrane bound in a complex with Scp1 (SREBP cleavage activating protein)[8]. However, *A*. *fumigatus* contains no functional characterized Scp1[15]. Periods of low oxygen or decreased sterols trigger the cleavage of fungal SREBPs by Stp1 in *C*. *neoformans*, or by via mechanisms involving an ubiquitin ligase and other proteases *A*. *fumigatus* and *S*. *pombe*[8,11,16]. The N-terminal region is then released and binds to sterol regulatory elements (SRE) in the nucleus, inducing the expression of hypoxic-genes[7,8,11]. Unlike the other fungi, *S*. *pombe* contains another regulatory mechanism in the nucleus. When O_2_ levels are high Ofd1 binds to Sre1 targeting it for proteasomal degradation; and when O_2_ levels are low Ofd1 transcription and protein levels increase, but is inhibited in the nucleus by the nuclear transporter, Nro1[15,17–22]. Although putative Ofd1 homologs are identified in *A*. *fumigatus* and *C*. *neoformans*, they do not serve the same function[13,15].

Mammalian SREBPs share common features with fungal SREBPs, and function to regulate the expression of cholesterol uptake, cholesterol synthesis and lipid metabolism genes[23]. Like fungi, mammalian SREBPs require cleavage from the cell membrane for activation and subsequent induction of downstream gene targets[23]. However, activation is sterol dependent, and cleavage from the cell membrane is inhibited when sterols levels are adequate. Mammalian SREBPs directly activate the expression of more than 30 genes required for lipid (LDL receptor and lipoprotein lipase genes) and sterol (Erg family of genes) metabolism, but have not been shown to be influenced by changes in oxygen levels [23–27].

The response to hypoxia in *H*. *capsulatum* is the transcriptional induction of its SREBP, *Srb1* together with the hypoxia-responsive genes (HRGs), *NADP/FAD*, *ABC*, *NIT50* and *RSP/GEF*. In addition, the genes that encode C-8 sterol isomerase *(Erg2)* and C-5 sterol desaturase *(Erg3)* increase under hypoxia in a time-dependent manner[28]. Unlike the other fungi studied, *H*. *capsulatum* is a dimorphic fungus that is internalized by the hosts’ immune cells and infection results in the formation of a hypoxic granuloma. Despite the biological importance of oxygen to *H*. *capsulatum*, little information exists regarding the processes underlying hypoxia tolerance in this dimorphic fungus.

This study is focused on characterizing the function of *Srb1* in *H*. *capsulatum* in order to advance our understanding of how *H*. *capsulatum* adapts to hypoxia. We postulated that *H*. *capsulatum Srb1* may similarly control adaptation to hypoxia and the hypoxic response may be essential for fungal growth and pathogenesis. We report here that *Srb*1 is necessary for *H*. *capsulatum* survival under hypoxia, itraconazole susceptibility and is required for fungal virulence.

## Results

### *Srb1* is necessary for *H*. *capsulatum* survival under hypoxia

**Fig 1:**
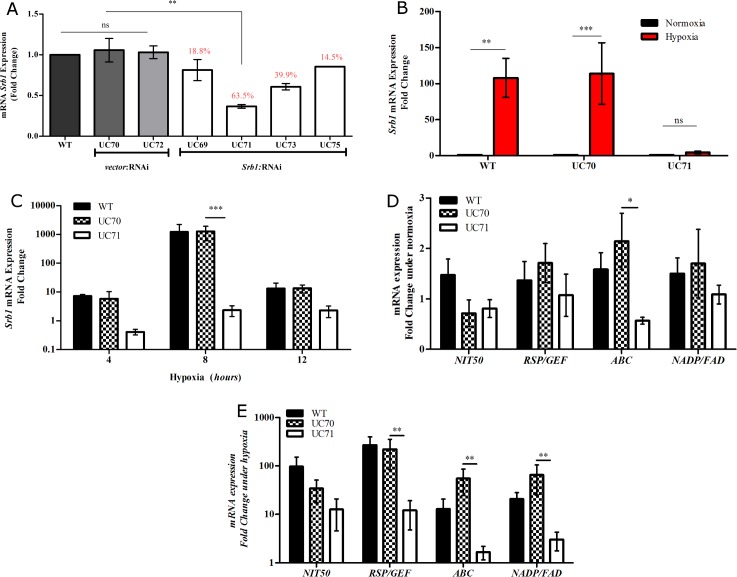
RNAi triggers effective silencing *Srb1* in *H*. *capsulatum*. (**A)**. Quantitative RT-PCR analysis of *Srb1* demonstrates a 14.5% to 63.5% reduction in *Srb1* transcript levels in characterized *Srb1*-RNAi strains, but no reduction in wild-type-vector strains. RNA was extracted from yeast grown in normoxia for 72 hours.**(B)** *Srb1* expression is not induced after 8 hours of hypoxia in *Srb1* silenced *H*. *capsulatum*, UC71 (*Srb1*:RNAi), compared to wild-type and wild-type-vector strain, UC70 (*vector*:RNAi). RNA was extracted from yeast grown under hypoxia for 8 hours. **(C)** *Srb1* expression remained at basal levels after 4, 8 and 12 hours of hypoxia when *Srb1* is silenced. RNA was extracted from yeast grown in hypoxia for 4, 8 or 12 hours. **(D)** *ABC* and *RSP/GEF* expression is significantly lower in UC71 (*Srb1*:RNAi) compared to WT and UC70 (*vector*:RNAi) under normal atmospheric conditions. RNA was extracted from yeast grown in normoxia for 72 hours. **(E)** *ABC*, *RSP/GEF*, *NADP/FAD* expression are not significantly induced in UC71 (*Srb1*:RNAi) after 8 hours of hypoxia. RNA was extracted from yeast grown in hypoxia for 8 hours. For all panels above RNA was extracted from wild type and silenced strain yeast grown under conditions described. Transcript levels were normalized to the constitutively expressed GAPDH gene. Data are represented as the mean ± standard error mean (SEM) of 3–4 experiments (Panel A) and 2–3 experiments (Panels C-E).

**Fig 2:**
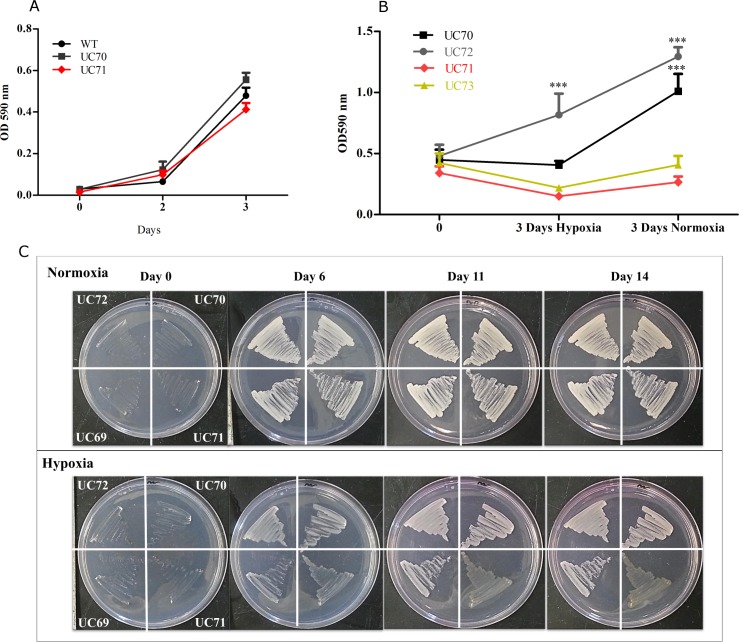
*H*. *capsulat*um *Srb1* expression is required for hypoxic growth. **(A)** Growth curve of WT, UC70 (*vector*:RNAi) and UC71(*Srb1*:RNAi), under normoxic conditions. **(B)** Growth curve of UC70 and UC71 in liquid HMM after 3 days of hypoxia followed by an additional 3 days of normoxia. Yeasts were inoculated with 5x10^6^ /ml of HMM. (**C)** Growth pattern of wild-type-vector strains UC70 and UC72, and *Srb1*:RNAi strains UC71 and UC69 streaked onto HMM plates supplemented with blasticidin and cultured for 14 days. (**D)** After 11 days of hypoxia HMM plated yeasts were placed under normoxia for an additional 11 days *(i)* or re-streaked and placed under hypoxia *(iii)* or normoxia *(ii)* for 11 days. Data shown are the results of 3–4 experiment and represented as mean ±SEM.

To determine whether *H*. *capsulatum* requires *Srb1* for its survival under hypoxia, we silenced the gene encoding *Srb1* using RNA interference (RNAi) in the wild type strain, G217B. The telomeric plasmid shuttle vector targeting *Srb1* for degradation, was constructed based on the predicted encoding *Srb1* gene sequence, and vectors were transformed into *H*. *capsulatum* as previously described [29], S1 Fig. This method resulted in a reduction in *Srb1* expression ranging from 14% to 63%, rigorously confirmed by qRT-PCR, Fig 1A. The *Srb1*:RNAi strains were named UC69 (18.8% knockdown), UC71 (63.5% knockdown), UC73 (39.9% knockdown) and UC75 (14.5% knockdown), and two wild-type-vector strains transformed with the vector only *vector*:RNAi, designated UC70 and UC72. Wild-type-vector strain UC70 and UC71 (silenced by 63.5%) were selected for extensive analysis. Both strains displayed normal growth rates and morphology over 3 days of culture under normoxic conditions, compared to wild type strain G217B in Histoplasma Macrophage Media (HMM), Fig 2A.

### *Srb1* is required for the expression of other hypoxia-responsive genes

*H*. *capsulatum* induces the expression of 4 key gene transcripts: *NIT50* (DQ350702), an *ABC* transporter (XM_001544189), a *NADP/FAD* oxidoreductase (XM_001544225) and an *RSP/GEF* (XM_001539669), after 24 hours of *in vitro* hypoxia within a hypoxia chamber [28]. Therefore, we sought to determine whether *Srb1* influenced the upregulation of these hypoxia-responsive genes (HRG). We first evaluated the levels of *Srb1* mRNA expression under hypoxia when *Srb1* is silenced by comparing UC71 to wild-type and wild-type-vector strain, UC70. When cultured under hypoxia for 8 hours, the period when *Srb1* expression is highest in *H*. *capsulatum*, there is an insignificant increase in *Srb1* in UC71 compared to wild- type (G217B) and UC70, Fig 1B. This defect in *Srb1* upregulation under hypoxia is detected as early as 4 hours after hypoxia treatment and maintained after 12 hours, Fig 1C. This data confirms that RNAi silencing technology was not only able to suppress *Srb1* expression under normoxia, but also maintain gene silencing under hypoxia.

To determine the influence of *Srb1* silencing on hypoxia-responsive genes, the basal HRG expression of UC71 was compared to wild-type and UC70 under normoxia, and also after 8 hours of hypoxia compared to normoxia. Quantitative RT-PCR (qRT-PCR) analysis of HRGs showed that under normal atmospheric conditions of ~21% O_2_, only *ABC* mRNA expression was significantly lower in *Srb1* silenced strain UC71, compared to UC70, Fig 1D. Suggesting that unlike the other HRGs, *ABC* mRNA is strongly regulated by *Srb1* and can influence its transcription even under normoxia. As previously reported, *NIT50*, *ABC*, *NADP/FAD* and *RSP/GEF* were significantly upregulated during hypoxia in wild-type and wild-type-vector strains[28]. However, when *Srb1* is silenced, *ABC*, *NADP/FAD* and *RSP/GEF* expression was significantly lower than wild-type-vector UC70, after 8 hours of hypoxia, Fig 1E. Though an increase in *ABC* mRNA was observed in UC70 compared to wild-type under conditions of both normoxia and hypoxia, this difference was not found to be statistically significant, Fig 1D and 1E. This strongly suggests that *Srb1* is involved in the regulation of HRG mRNA expression and these genes may be potential downstream targets of the transcription factor.

While we have demonstrated that *H*. *capsulatum* remains viable under O_2_ concentrations of less than 1% [28], we sought to determine the effect of *Srb1* silencing on *H*. *capsulatum* during periods of extended hypoxia. Optical density, used to quantify culture turbidity, was used as a measurement of yeast growth. Compared to *H*. *capsulatum* growth before hypoxia (time-point 0), after 3 days of hypoxia (1% O_2_, 5% CO_2_, 94% N2), there was no change in wild-type-vector strain UC70, while there was a significant increase in yeast concentration in wild-type-vector strain UC72. In contrast, hypoxia resulted in the killing of *Srb1* silenced yeasts UC71 and UC73, silenced by 63.5% and 39.3%, respectively, Fig 2B. When transferred back to oxygen replete conditions after hypoxia incubation, UC70 and UC72 replenished growth, while no growth of UC71 and UC73 was observed, Fig 2B. Our lab has previously demonstrated that wild type (G217B) *H*. *capsulatum* growth is inhibited under hypoxia and displays visual differences in colony size, but not colony number when compared to normoxia [28].

As a secondary method to determine the phenotype of *H*. *capsulatum* when *Srb1* is silenced, yeast strains were streaked onto solid agar plates to compare colony growth under normoxia to hypoxia. After 11 days of incubation under hypoxia, wild-type-vector strains UC70 and UC72 showed a visible increase in yeast density, while UC71 continued to show limited or no growth and changed to a brownish color, Fig 2C. Not surprisingly, strain UC69 silenced by only 18.8%, was able to survive 11 days of hypoxia, as demonstrated by the noticeable increase in yeast density, Fig 2C.

In a third experiment, yeast strains cultured on solid agar under hypoxia for 11 days, were placed in normoxia for an additional 11 days. UC71 growth remained impaired compared to wild-type-vectors, UC70 and UC72, Fig 2D *(ii)*. Strain UC75 (14.5% knockdown) however, continued to replicate and was unaffected by hypoxia much like UC69 (18.8% knockdown), Fig 2D *(ii)* and 2C.

In parallel, each strain from Fig 2D *(i)* (11 days of hypoxia), was re-streaked and placed under normoxia or hypoxia for an additional 11 days, Fig 2D *(iii)* and *(iv)*, respectively. In congruence with growth in liquid HMM, wild-type-vector strains, UC70 and UC72 were able to replenish growth after replacement under normoxia, however only minimal growth was observed in *Srb1* silenced strain UC71, Fig 2D *(iii)*. In contrast, after 11 days of hypoxia, wild-type and wild-type-vector strains continued to replicate, while there was no visible growth in UC71, Fig 2D *(iv)*. UC75 maintained a phenotype similar to that of wild-type-vector strains under both conditions. Taken together these data demonstrate that *Srb1* silencing has an irreversible effect on *H*. *capsulatum’s* ability to recover from hypoxia. *H*. *capsulatum* can quickly adapt to hypoxia, and *Srb1* is involved in mediating this response by an undefined mechanism.

### *Srb1* silencing inhibits the expression of the ergosterol biosynthetic pathway genes during hypoxic adaptation

**Fig 3:**
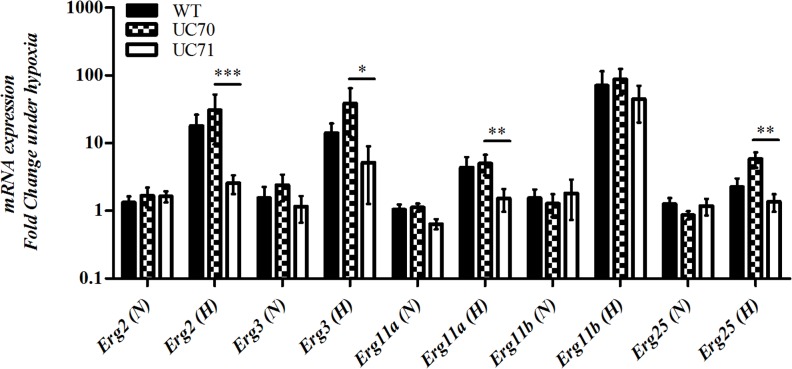
*Srb1* regulation of ergosterol biosynthesis genes during hypoxia. Expression of ergosterol pathway genes *Erg2*, *Erg3*, *Erg11*, *and Erg25* under normoxia compared to 8 hours of hypoxia. Values in parenthesis represent (N) normoxia and (H) hypoxia. Data represent the means and SEM of 3 experiments.

To determine if *Srb1* is a regulator of genes involved in the ergosterol biosynthetic pathway, we examined the effect of *Srb1* silencing on the mRNA expression of genes encoding the enzymes Erg2, Erg3, Erg11 and Erg25 in yeast grown in normoxia compared to 8 hours of hypoxia. These enzymes needed for ergosterol biosynthesis, all require oxygen for their function[30]. There are 2 paralogs of the *Erg11* gene of which have been designated *Erg11a* and *Erg11b*. Fig 3 shows that upon switching to hypoxia for 8 hours, *Erg2*, *Erg3*, *Erg11a*, *and Erg25* was significantly upregulated in wild-type and UC70, but not UC71 cells. Expression of these genes under normal atmospheric conditions was only slightly reduced in UC71 compared to UC70, therefore basal expression was not significantly affected, Fig 3. Interestingly, *Srb1* had no effect on *Erg11b* expression, and its expression remained comparable between all strains under both conditions. This suggests that *Srb1* is required for the maximum induction of *Erg2*, *Erg3*, *Erg11a* and *Erg25*, and that as in mammals, may also have an essential role in regulating the genes necessary for ergosterol biosynthesis.

### Loss of *Srb1* results in impaired growth *H*. *capsulatum* within macrophages

To examine the role of *Srb1* during *H*. *capsulatum* infection, we tested the ability of *Srb1* depleted yeast cells to replicate within macrophages. Bone marrow derived murine macrophages (BMDMs) were infected with wild-type, control vector (UC70) and *Srb1* silenced *H*. *capsulatum* strains (UC71, UC75) at a multiplicity of infection (MOI) of 0.1 and 1. Two (2) hours following co-incubation, BMDMs were washed with fresh media and either lysed or allowed to incubate for a total of 24 hours. At both 2 and 24 hours post infection, macrophages were lysed and plated to quantify the remaining viable yeast by determining the number of colony-forming units (CFUs).

**Fig 4:**
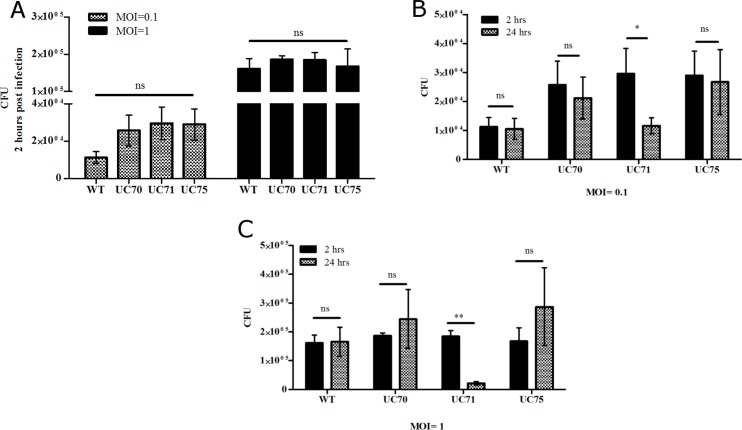
Loss of *Srb1* reduces *H*. *capsulatum* survival within murine macrophages. **(A)** An equal number of colony-forming units (CFU) recovered from bone marrow derived macrophages (BMDM) after 2 hours of infection with WT, UC70 or UC72. Chart depicts CFU after 24 hours post infection at a multiplicity of infection (MOI) of **(B)** 0.1 and **(C)** 1. Results represent the mean ± SEM of 4 independent experiments.

Fig 4A shows that the rate of phagocytosis between strains UC70, UC71, UC75 was similar to wild-type, as equal number of yeasts cells were phagocytosed by the macrophages, observed after 2 hours of infection. After 24 hours, both wild-type (WT) and wild-type-vector (UC70) strains survived within the BMDM. However, there was a 60% and 85% decrease in the number of viable UC71 yeast cells within macrophages after 24 hours at MOIs of 0.1 and 1, respectively, Fig 4B and 4C. Strain UC75 (silenced by 14.4%) demonstrated no change in viability after 24 hours within BMDMs compared to 2 hours post infection. Thus, 14.4% silencing of *Srb1* was not sufficient to adversely affect the ability of *H*. *capsulatum* to thrive within BMDMs.

By using this *in vitro* assay for survival within macrophages, a mutant was identified that possesses a diminished capacity for intracellular macrophage replication. Wild-type and wild-type-vector (UC70) *H*. *capsulatum* were able to survive within the macrophages. However, there was a defect in *H*. *capsulatum* proliferation within BMDMs when *Srb1* was silenced by 63.5% (UC71), Fig 4B and 4C. Thus, without *Srb1* expression, *H*. *capsulatum* is not able to withstand the intracellular environment of a macrophage.

### *Srb1* is required for *H*. *capsulatum* virulence

**Fig 5:**
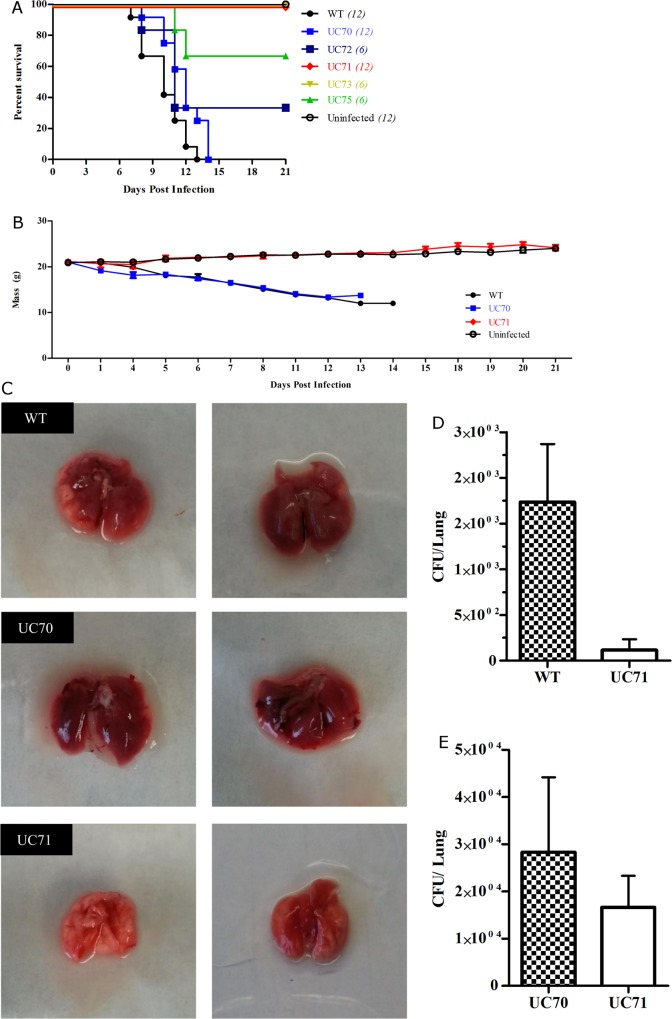
*Srb1* is required for the virulence of *H*. *capsulatum* yeasts. **(A)** Six (6) male C57B/6 mice per group were intranasally infected with a lethal dose of 2 x 10^7^ WT, UC70 or UC71 yeasts and observed for 21 days. Experiments were performed twice and data from the total of twelve mice were combined where indicated in parenthesis. (**B)** Graph represents mouse weights taken until death or sacrifice. **(C)** Gross pathology of mouse lungs infected with WT, UC70 or UC71 taken 14 d.p.i with 2x10^7^ yeasts. **(D)** Fungal burden of four (4) mouse lungs 14 days post lethal (2x10^7^) yeasts. **(E)** Fungal burden of four (4) mouse lungs 7 days post sub-lethal (2 x10^6^) infection. Values in parenthesis represent total number of mice. Long rank (Mantel Cox) test was performed on survival curve *** P< 0.0001.

Infectivity studies were undertaken to examine the virulence of *Srb1* silenced *H*. *capsulatum*, and to determine its importance during the course animal infections. To assay virulence, we inoculated groups of 6 C57BL/6 mice with a lethal dose (2×10^7^) of either wild-type, wild-type-vector (UC70, UC72), or *Srb1* silenced strain (UC71, UC73, UC75), intranasally. Wild-type and UC70 challenged mice succumbed to infection by day 13 and 14 post infection, respectively, Fig 5A. In contrast, all mice challenged with either UC71 or UC73 (39.9% knockdown) survived through three weeks, showed no adverse symptoms and surpassed their initial body weight, Fig 5A and 5B. Four (4) out of six (6) mice inoculated with wild-type vector UC72, succumbed to infection by day 10; while two (2) out of six (6) mice infected with UC75 (14.5% knockdown) also died by 12 d.p.i. Macroscopically the lungs of mice infected with the UC71 *Srb1* silenced strain, showed almost no signs of infection and appeared pink and spongy. While lungs of mice challenged with either UC70 or wild-type strains were dark red and firm with the appearance of lesions, Fig 5C. The corresponding CFU cultured from four (4) mouse lungs inoculated with UC71 was remarkably lower than those challenged with wild-type *H*. *capsulatum*, Fig 5D. In a separate single experiment we observed a decrease in lung fungal burden in mice infected with a sublethal dose (2x10^6^) of UC71 compared to UC70 at 7 d.p.i., Fig 5E. This data strongly supports the theory that *Srb1* is required for *H*. *capsulatum* virulence in mice.

### *Srb1* silencing confers *H*. *capsulatum* sensitivity to itraconazole

**Fig 6:**
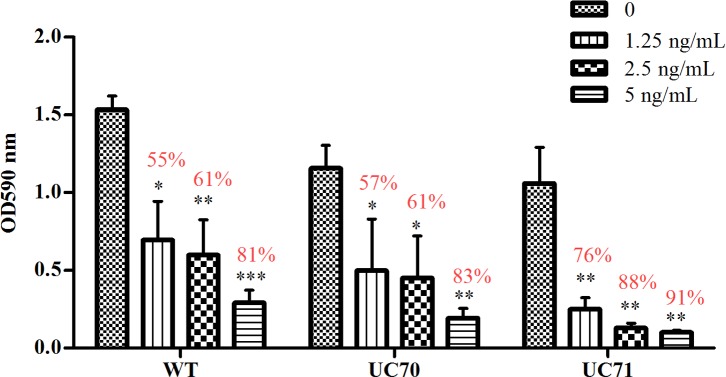
*H*. *capsulatum* susceptibility to itraconazole when *Srb1* is silenced. Graph represents a comparison of wild-type, wild-type-vector (UC70) and *Srb1*:RNAi (UC71) survival in different concentrations of itraconazole. OD600 measurement after 6 days in culture was used to quantify yeast growth. Values in red represent the percentage difference in growth compared to no treatment group (0). Statistical analysis conducted on 3 experiments represent the mean ± SEM of OD600 absorbance of *H*. *capsulatum* in each itraconazole concentration compared to no treatment group.

We have found that without *Srb1* expression, induction of 4 oxygen-requiring ergosterol biosynthesis genes (*Erg 2*, *Erg3*, *Erg11a* and *Erg25*) was impaired during hypoxia. Therefore, we sought to determine whether *Srb1* silencing affected the ability of the ergosterol synthesis inhibitor, itraconazole to exert its fungistatic effects on *H*. *capsulatum*. Itraconazole targets the ergosterol pathway in fungi particularly the product of the Erg11 gene. Erg11 encodes lanosterol 14-alpha-demethylase, the enzyme that converts lanosterol to ergosterol[31]. We cultured *Srb1* silenced strain UC71, WT and wild-type-vector strain UC70 with varying concentrations of itraconazole and measured their growth by culture optical density after 6 days. A concentration of 1.25 ng/ml of intraconazole inhibited wild-type and wild-type-vector growth by 55 and 57% respectively. Surprisingly, this concentration was enough to inhibit UC71 growth by 76%, Fig 6. With 2.5 ng/ml of itraconazole, both wild-type and UC70 growth was inhibited by 61%, while UC71 growth was inhibited by 88%. At 5 ng/ml of itraconazole, a concentration known to completely inhibit *H*. *capsulatum*, there was an 81% (wild-type), 83% (UC70) and 91% (UC71) decrease in growth compared to control not treated with itraconazole. Silencing *Srb1* dramatically increases the susceptibility of *H*. *capsulatum* to itraconazole. The concentration of drug needed to inhibit growth is much lower when *Srb1* is silenced. This susceptibility phenotype is similar to that observed with SrbA mutants in *A*. *fumigatus* and Sre1 mutants in *C*. *neoformans*[32,33].

In Fig 3, we examined the mRNA abundance of *Erg11a* and *Erg11b* transcripts when *Srb1* is silenced, and a statistically significant decrease in mRNA abundance was observed in *Erg11a* mRNA UC71 compared to wild type. *Erg11b* mRNA abundance was also consistently higher in the Srb1 silenced strain than *Erg11a*. Thus, these results suggest that a decrease in the *Erg11a* transcript in the UC71 strain may confer the increased susceptibility of this strain to itraconazole and that *Srb1* may directly regulate *Erg11a* transcript abundance.

## Discussion

The mechanisms that underlie *H*. *capsulatum* gene regulation under hypoxia are complex and intricate. While we have identified a role for *Srb1* in this pathway, much remains to me elucidated and studied. Our studies provide evidence that the *H*. *capsulatum* sterol regulatory element binding protein, *Srb1* is crucial for adaptation to hypoxia in *H*. *capsulatum*. When *Srb1* expression is silenced by both 63.5% and 39.9%, *H*. *capsulatum* cannot survive under oxygen conditions of less than 1%, and is incapable of replenishing growth under normoxia. However, silencing by 18.8% or 14.5% did not result in measurable changes in *H*. *capsulatum* growth, Fig 2. Thus, the ability of *H*. *capsulatum* to survive hypoxia was dose dependent on the degree of silencing of *Srb1;* and effective silencing of *Srb1*, of more than 39.9%, resulted in a strain that was defective in growth under conditions of hypoxia, but unaffected when under normal atmospheric oxygen conditions (~21% O_2_). In eukaryotes, control of metabolic processes becomes critical for survival during microenvironmental stress conditions; and under low-oxygen conditions sterol, fatty acids, iron, and heme homeostasis can be severely dysregulated[11,16,30,34–37]. This data shows that in order to exert its function, more than 60% of *Srb1* expression is necessary for *H*. *capsulatum* to survive hypoxia.

Other key players in the hypoxia response network include an *ABC* transporter, an *NADP/FAD* oxidoreductase, an *RSP/GEF and NIT50*. These genes were previously reported to be significantly upregulated under hypoxia in *H*. *capsulatum*, but are yet to be functionally characterized[28]. In this study, upregulation was once again examined under hypoxia and data presented at an 8 hour time point, the timing of maximal upregulation, although smaller amplitude up regulation was noted at 2 and 4 hour timepoints. The 8 hour timepoint may reflect maximal upregulation but may be influenced by factors other than hypoxia such as nutritional deprivation. When *Srb1 i*s silenced hypoxic induction of *ABC*, *NADP/FAD* and *RSP/GEF*, but not NIT50, was inhibited, Fig 1D and 1E. In other eukaryotic organisms, these putative *H*. *capsulatum* proteins are known to be involved in the transport of metabolic products, lipids and sterols, and drugs (ABC); ergosterol biosynthesis associated and coordinately regulated with Erg11p (NADP/FAD), responsive to nitrosative stress (NIT50) and guanine nucleotide exchange (RSP/GEF) [5,31,38–43]; all processes essential for basic metabolism. Many of those molecules whose biosynthesis is increased under hypoxia, are likely indirect sensors of oxygen that orchestrate changes in overall fungal metabolism to allow *H*. *capsulatum* to adapt to hypoxia[13]. This data supports the hypothesis that *Srb1* is involved in the regulation of basic *H*. *capsulatum* metabolic processes during hypoxic stress. Nonetheless, how *H*. *capsulatum* senses and responds to changing oxygen levels remains an area of investigation.

We provide evidence to support Srb1 as the functional homolog of fungal SREBP, and SREBPs are known to regulate the expression of many genes in different eukaryotes. Mammalian SREBP and *C*. *neoformans* Sre1 are transcriptional activators that regulate expression of every sterol biosynthetic enzyme [8,44]. Whereas *S*. *pombe* Srel and *A*. *fumigatus* SrbA do not control the entire sterol biosynthetic pathway, but predominantly enzymes involved in oxygen-requiring reactions[7,10]. Here, our initial analysis of *Srbl*-dependent gene expression indicates that *H*. *capsulatum Srbl* controls expression of genes required for the oxygen-dependent reactions (ERG2, ERG3, ERG11 and ERG25). *Srb1* as a transcriptional regulator may regulate genes under conditions other than hypoxia but the current studies examined only this experimental condition.

Ergosterol is a critical part of the fungal cell membrane and inhibition of its synthesis results increases cellular permeability causing leakage of its cellular contents[45]; and as the name suggests, *Srb1* may also be essential for ergosterol homeostasis in *H*. *capsulatum*. We found that *Srb1* silencing resulted in a strain of *H*. *capsulatum* that was more sensitive to the antifungal drug, itraconazole. Yet the mechanism for the increased susceptibility and whether this fungal SREBP mediates ergosterol biosynthesis remains unknown. Itraconazole interacts with 14-α demethylase (ERG11), a cytochrome P-450 enzyme that converts lanosterol to ergosterol [8,13]. The putative *NADP/FAD*, cytochrome P-450 reductase is also associated and coordinately regulated with Erg11p[31]. This increased sensitivity is likely to be due to the decreased expression of *Erg11a* and *NADP/FAD* in the absence of *Srb1*.

Silencing *Srb1* expression in *H*. *capsulatum* allowed us to study whether it is necessary for virulence and progression of murine histoplasmosis. Disrupting *Srb1* expression resulted in an avirulent *H*. *capsulatum* strain that cannot effectively replicate within the macrophage and is not pathogenic in mice. The *H*. *capsulatum* strain silenced by 63.5% (UC71), exhibited enhanced susceptibility to killing by macrophages compared to wild-type. It is well established that once internalized *H*. *capsulatum* is able to replicate within unactivated macrophages[46–48], but we were not able to detect a significant increase in wild-type yeast replication within BMDMs, Fig 4B and 4C. The lack of replication if wild-type yeast may be related to the derivation of macrophages by using GM-CSF to differentiate bone-marrow monocytes, as GM-CSF, if incubated with monocytes for extended periods can stimulate macrophage fungistatic activity and inhibit *H*. *capsulatum* replication[49].

Mice infected with UC71 showed no signs of illness and survived until sacrifice at 21 d.p.i. while wild-type infected mice died by day 14. In addition, lungs extracted from UC71 infected mice appeared healthy compared to wild-type and wild-type-vector infected mice. It is possible that this virulence defect in *Srb1* silenced strains is due to the presence of hypoxic microenvironments in host tissue during infection, in which *H*. *capsulatum* is not able to adapt. Though not tested, we postulate that without *Srb1* there is a decrease in ergosterol production, necessary for cell wall and membrane maintenance as a result of differences in *Srb1*-dependent gene expression between wild-type and UC71.

While *Srb1* appears to be a master regulator of hypoxia in *H*. *capsulatum*, we also hypothesize that a complex network of proteins and enzymes may also be necessary for adaptation to hypoxia. Ofd1 regulates both DNA binding and the degradation of Sre1 in *S*. *pombe* in an oxygen-dependent manner. In the presence of oxygen, Ofd1 binds to Sre1 and keeps Sre1 from binding to DNA by accelerating Sre1 degradation [10,50]. During low oxygen tension, Ofd1-Sre1 binding is inhibited by Nro1, slowing down Sre1 degradation and allowing it to bind to DNA and upregulate hypoxic gene transcription [17,19]. An *Ofd1* homolog identified in H. capsulatum may be one such interactive proteins and its expression is upregulated under hypoxia (data not shown). The transcriptional regulation and role of *Ofd1* in the hypoxic response differs between *S*. *pombe* and *A*. *fumigatus* and *C*. *neoformans* [51].These studies indicate that the function of *Srb1* as a hypoxic transcription factor is conserved across fungi in both ascomycetes (*S*. *pombe*, *A*. *fumigatus*) and a basidiomycete (*C*. *neoformans*). Our data provides evidence that *H*. *capsulatum*, a dimorphic intracellular pathogen, has evolved a complex pathway to survive not only nutrient and mineral deficiencies but also changes in oxygen levels. The mechanisms that underlie *H*. *capsulatum* gene regulation under hypoxia are complex and intricate. While we have identified a role for *Srb1* in this pathway, much remains to be elucidated and studied.

## Materials and Methods

### Strains and Culture Conditions

All *Histoplasma capsulatum* strains utilized in this study were derived from the ATCC *Histoplasma capsulatum* wild-type strain G217B (ATCC # 26032) and are listed in Table 1. *H*. *capsulatum* strains were maintained on Histoplasma-Macrophage Medium (HMM) [52], at 37°C with 5% CO_2_. For solid medium HMM was solidified with 0.8% agarose (Lonza SeaKem ME, Pittsburgh, PA, USA) and supplemented with 25 M FeSO4. Liquid cultures were grown until log phase in an orbital shaker with continuous shaking for 72 hours at 200 rpm. For growth of blasticidin resistant strains, plates and media were supplemented with 100 μg/mL blasticidin S (Invivogen, San Diego, CA, USA) where appropriate. Normoxic conditions were deemed general atmospheric levels within the incubator (~21% O_2_). Hypoxic conditions (<1% O_2_), were achieved using a controlled atmospheric chamber (BioSpherix C-Chamber, Winston-Salem, NC, USA) with O_2_ and CO_2_ levels controlled by an external controller. The chamber was maintained at 37°C and kept at ~1% O_2_ utilizing a gas mixture of 94% N_2_ and 5% CO_2_. Growth rates of RNA interference (RNAi) yeast strains were determined in liquid media, by measurement of culture turbidity at an absorbance of 590 or 600 nm. Precise enumeration of yeasts was done by hemocytometer counts.

**Table 1:** *H*. *capsulatum* strains.

Strain	Genotype	Other names
G217B	Wild Type	
UC70	G217B pSK-Tel-Kan-Blast-186	*vector*: RNAi #1, wild-type-vector
UC72	G217B pSK-Tel-Kan-Blast-186	*vector*: RNAi #2, wild-type-vector
UC69	G217B pSK-Tel-Kan-Blast-186-*Srb1*	*Srb1*: RNAi #1
UC71	G217B pSK-Tel-Kan-Blast-186-*Srb1*	*Srb1*: RNAi #2
UC73	G217B pSK-Tel-Kan-Blast-186-*Srb1*	*Srb1*: RNAi #3
UC75	G217B pSK-Tel-Kan-Blast-186-*Srb1*	*Srb1*: RNAi #4

For itraconazole experiments, all yeast strains were cultured under normoxia within a shaking incubator at 37°C for 6 days. Yeasts were grown in media containing HMM supplemented with 1.25, 2.5, and 5 ng/ml of intraconazole dissolved in DMSO; and HMM with DMSO served as the no treatment control. Optical density was used to as a measure of yeast growth and experiments were conducted in triplicate.

### Strain Construction

#### *UC69*, *UC71*, *UC73 and UC75 (Srb1*:RNAi)

*H*. *capsulatum Srb1*:*RNAi* strains were constructed in G217B wild-type strain using a hairpin-loop telomeric silencing plasmid to knockdown the expression of *Srb1* (S1 Fig). An *H*. *capsulatum* shuttle vector pSK-Tel-Kan-Blast-186 was previously constructed by fusion of the pSKII+ backbone containing the origin of replication and multiple cloning sites, with a fragment from pCR83 containing *H*. *capsulatum* telomere sequence repeats flanking the kanamycin resistance cassette, a fragment containing the *Aspergillus terreus* blasticidin deaminase gene *BSD* under control of the *gpd* promoter flanked by the *Aspergillus nidulans trpC* terminator, and a fragment of pCR186 (a kind gift from Chad Rappleye) containing a *gfp* silencing cassette under the control of the *H*. *capsulatum pH2AB* promoter[53,54]. Restriction endonuclease digestion and ligation was used to replace the *gfp* gene with *Srb1*. A 470 bp fragment of the *Srb1* coding region with compatible restriction site end sequences, was generated by PCR and cloned into the plasmid constructs in forward and reverse directions using relevant primers Table 2. The resulting plasmid containing the *Srb1* cDNA under control of the H2AB promoter was linearized through *PacI* restriction digestion and transformed into *H*. *capsulatum* G217B by electroporation under standard conditions[55]. Transformants were subsequently cultured in liquid HMM media for 24 hours then passaged in HMM supplemented with 100 μg/mL blasticidin S and plated on HMM-blasticidin solid media. Individual colony transformants were replica plated onto HMM-blasticidin. Plasmid transformed yeast were selected based on resistance to the antibiotic blasticidin S due to the presence of the plasmid encoding blasticidin resistance gene, *BSD*, which confers resistance in *H*. *capsulatum*. The loss of the *srb1* gene was verified by quantitative Real-Time PCR (qRT-PCR) using *Srb1* specific primers in clones only able to grow in the presence of blasticidin S.

**Table 2:** List of *H*. *capsulatum* primers used.

***PRIMERS***	**SEQUENCE**
***SRB1***	S: GTAGCAGCCGAACAACATCTG
	AS: AATGAGACCTTGGGCGATACG
***NIT50***	S: CGCCACTTCAACAACACCA
	AS: GCCTTTCCAGTCCGCTTTCA
***RSP/GEF***	S: CGTCACTCATCAATCCACGG
	AS: AATCCTGTCCTGCCTCTTGG
***ABC***	S: GCGACACAAATGAACACAGAC
	AS: AGCAACATCAACATCACCCG
***NAD/FAD***	A: TGTCACTGTCCACGAATCCC
	AS: TCAAACCACGGAACATCGGG
***Erg2***	S: TTCAGCAACCACGGAAAC
	AS: CGCCATGCAGTATCGTAAA
***Erg3***	S: GGATTATGCCAAGCCCTTAC
	AS: CAGGACAGTCCAGATGTTAATG
***Erg11a***	S: TTCTTGGAACAAAAGGCAACG
	AS: CGAGGTTAGCCCGTATTTGAC
***Erg11b***	S: CTATGGAACCGACCCGTATAAG
	AS: TCGTTGCCCTTTATGCCTAG
***Erg25***	S: GCAATAAAATCCCTAGCCTGAAG
	AS: TTTGATAAGTCATAGTCCACGGG
***Gapdh***	S: ATTGGGCGTATTGTCTTCC
	AS: TTGAGCATGTAGGCAGCATA
***VECTOR CONSTRUCTION PRIMERS***	SEQUENCE ^a^
***Srb1f-asc1***	S: T GGCGCGCC AGCAAAGCATCCGACTAAG
***Srb1f-xho1***	AS: T CTCGAG TTTCAACATCACCCGTGC
***Srb1r-spe1***	S: T ACTAGT AGCAAAGCATCCGACTAAG
***Srb1r-age1***	AS: T ACCGGT TTTCAACATCACCCGTGC

^**α**^ Underlined text represent restriction enzyme sequence.

#### UC70 and UC72 (Wild-type-vectors)

Using the same protocol described above the *H*. *capsulatum* shuttle vector pSK-Tel-Kan-Blast-186 was constructed without the *Srb1* silencing cassette. The empty vector plasmid was linearized with PacI restriction endonuclease, electroporated into *H*. *capsulatum* G217B and cultured in HMM for 24 hours[54]. Transformants with plasmid vectors were selected based on their ability to grow on HMM plates containing 100 μg/mL blasticidin S.

### Yeast RNA isolation and Quantitative Real-Time PCR

*H*. *capsulatum* yeast cells were collected from 5 mL HMM liquid cultures by centrifugation for 10 min at 1,600 rpm. Total RNA was extracted from fresh cell pellets using a MasterPure Yeast RNA Purification Kit (Epicentre Biotechnologies, Madison, WI, USA) following the manufacturer’s instructions, and treated with DNase I for removal of contaminating genomic DNA. RNA concentration and purity was determined using a NanoDrop ND-1000 spectrophotometer (ThermoScientific, Florence, KY, USA), at an absorbance ratio of 260 and 280 nm. cDNA was synthesized using 1 μg of RNA reverse transcribed using SuperScript II reverse transcriptase (Life Technologies, Grand Island, NY, USA), random primer mix and deoxynucleotide triphosphate (dNTPs). For qRT-PCR reactions templates were diluted 1:80 in a PCR mix containing each gene-specific primer pair and SYBR Green dye (Life Technologies, Grand Island, NY, USA). To ensure accuracy of cDNA amplification, at least one primer of each pair was generated to span an intron, and their inability to amplify genomic DNA was confirmed before use. Standard curves were generated for each primer set using 5-fold serial dilutions of pooled cDNA. Gene expression was measured on Applied Biosystems 7500 FAST PCR machine (Applied Biosystems, Foster City, CA, USA). For each sample, values obtained were normalized to the levels of glyceraldehyde 3-phosphate dehydrogenase (GAPDH), and RNA levels were calculated using the standard curve ddCT method. Oligonucleotide primers used for qRT-PCR study are listed in Table 2.

### Intranasal Mouse Infection

Six (6) week old C57BL/6 male mice purchased from Charles River Laboratories, Inc., (Wilmington, MA, USA) were used for infection. Mice were housed and maintained in micro-isolator cages in a barrier facility, under pathogen-free conditions, and supplied with sterilized food and water. All animal procedures were approved by the Institutional Animal Care and Use Committee of the Cincinnati VA Medical Center under protocol # 14-10-03-01. As described in the animal protocol, mice with histoplasmosis develop some level of distress manifest by ruffled fur, reduced activity and tachypnea. Animals infected with sub-lethal inoculum spontaneously recover from the infection over a 2–3 week period. Animals administered a larger lethal inoculum or animals with progressive infection manifest worsening tachypnea, severe hunching, and immobility. Animals were monitored at least twice each day to identify animals meeting a humane endpoint allowing sacrifice prior to spontaneous death. Mice with severe tachypnea, severe hunching or immobility in whom it was thought death may be imminent were euthanized. Euthanasia was performed using CO_2_ exposure in a gradual fill enclosure from a compressed CO_2_ cylinder followed by cervical dislocation as a secondary method. No analgesia was provided as it has not been deemed capable of relieving the distress seen with active infection. In the course of the experiments 25 of 185 animals died as a result of the Histoplasma challenge without sacrifice despite twice daily close monitoring. The death rate was higher than anticipated and that recorded in prior studies. All of these animals were determined to have died as a result of severe *Histoplasma* pneumonia.

Mice were infected intranasally (i.n.) with a lethal dose (2 x 10^7^) mid-exponential phase *H*. *capsulatum* yeast cells resuspended in 20 μL PBS. Groups of six (6) mice were infected with either G217B, UC70, UC71, UC72, UC73, UC75 strain. To avoid yeast clumping, all yeast cultures were vortexed prior to infection. The accuracy of infection was measured by determination of colony forming units of infection cultures (CFUs). Mice were weighed daily until 21 days post infection or until they succumbed to infection and were sacrificed.

In a separate experiment, lungs from four (4) lethally infected animals were removed aseptically before sacrifice 14 d.p.i., photographed and then homogenized for enumeration of fungal burden by CFU/lung, Fig 5D. For sublethal experiments, groups of 4 mice were infected intranasally (i.n.) with (2 x 10^6^) mid-exponential phase *H*. *capsulatum* yeast cells UC70 and UC71 resuspended in 20 μL PBS, Fig 5E. Lungs were removed, homogenized and plated on HMM at 7 d.p.i for the enumeration of surviving CFU.

### Macrophage Infection

Bone marrow derived macrophages (BMDM) isolated from C57BL/6 mice were infected with 5 x 10^5^ or 5 x 10^4^ mid- log phase *H*. *capsulatum* yeast cells in DMEM (Dulbecco's Modified Eagle Medium, Gibco, NY). BMDMs were harvested and cultured following standard protocol using GM-CSF[56]. Macrophages (5× 10^5^) were placed in 48-well plates and kept at 37°C in a 5% CO_2_ incubator overnight. Non-adherent cells were removed by aspiration and washing 3× with DMEM, and the plates kept at 37^0^ C in a 5% CO_2_ incubator. For infections, *H*. *capsulatum* yeast cells were grown to log phase in HMM or HMM with 100 μg/mL blasticidin S where appropriate. BMDMs were pelleted and washed 3x in sterile PBS, resuspended in DMEM supplemented with 10% fetal bovine serum, then vortexed twice for 5 seconds, and counted by hemocytometer. Macrophages were infected with *H*. *capsulatum* at a multiplicity of infection (MOI) of 1 yeasts per macrophage (1:1) or 0.1 yeasts per macrophage (0.1:1), yeast to macrophage ratio. Approximately 5 ×10^5^ (in 100 uL) *H*. *capsulatum* cells were used to infect 5 ×10^5^ BMDMs in 48-well cell culture dishes for 2 hours in DMEM supplemented with 10% fetal bovine serum, and gentamicin. After 2 hours non-adherent yeasts were removed by aspiration and the BMDM monolayer washed with DMEM 3x. Two groups of macrophages were tested as follows: a) to determine the number of yeasts phagocytosed after 2 hours the infected cells were harvested and lysed in sterile water by repetitive pipetting, b) fresh medium was added to each well for an additional 24 hours. After which, the culture medium was removed and the BMDM washed 3x with medium, then lysed in sterile water by repetitive pipetting. The lysates were centrifuged and yeasts were suspended in 1 mL of PBS or sterile water. Dilutions were plated onto HMM plates and incubated at 37°C for 1 week to determine CFUs.

### Statistical Analysis

All experiments were performed in duplicate or triplicate. All statistical analyses were performed using GraphPad Prism for Windows. The results are expressed as means ± S.E.M. The statistical significance between the control and experimental data was calculated using one-way or two way analysis of variance (ANOVA) with paired comparisons. A p-value of less than 0.05 was considered to be statistically significant. For mouse survival, log rank (Mantel Cox) test was performed on survival curves.

### Ethics Statement

All animal studies were carried out in accordance with the recommendations in the Guide for the Care and Use of Laboratory Animals of the National Institutes of Health and with the prior approval of the Cincinnati VA Medical Center Institutional Animal Care and Use Committee under protocol #14-10-03-01.

## Supporting Information

S1 Fig Schematic of the pSK-Tel-Kan-Blast-186 vector used to silence the *Srb1* gene in the wild-type G217B strain. Plasmid contains inverted copies of *Srb1* separated by a *lacZ* fragment to produce an RNA hairpin.
(TIFF)
