# Supplementary figures and images for: Sterol Regulatory Element Binding Protein (Srb1) Is Required for Hypoxic Adaptation and Virulence in the Dimorphic Fungus Histoplasma capsulatum
Source: PLoS One. 2016 Oct 6;11(10):e0163849. doi: 10.1371/journal.pone.0163849 (PMC5053422; doi:10.1371/journal.pone.0163849)

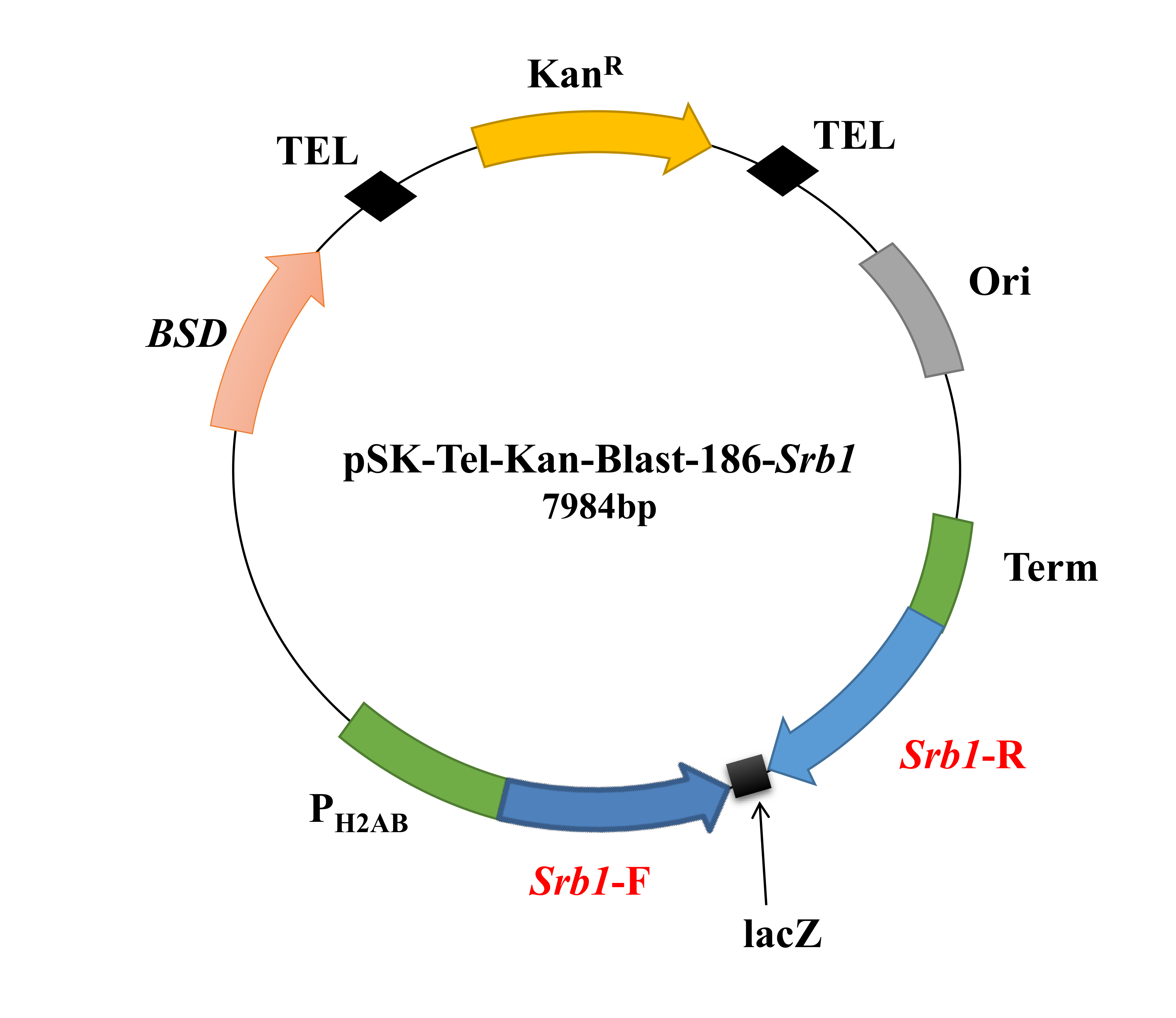

Supplement: S1 Fig — Plasmid contains inverted copies of Srb1 separated by a lacZ fragment to produce an RNA hairpin. (TIFF) [file pone.0163849.s001.tiff]
